# PIQMIe: a web server for semi-quantitative proteomics data management and analysis

**DOI:** 10.1093/nar/gku478

**Published:** 2014-05-26

**Authors:** Arnold Kuzniar, Roland Kanaar

**Affiliations:** 1 Department of Genetics, Cancer Genomics Netherlands, Erasmus Medical Center, PO Box 2040, 3000 CA Rotterdam, The Netherlands; 2 Department of Radiation Oncology, Erasmus Medical Center, PO Box 2040, 3000 CA Rotterdam, The Netherlands

## Abstract

We present the **P**roteomics **I**dentifications and **Q**uantitations Data **M**anagement and **I**ntegration Servic**e** or PIQMIe that aids in reliable and scalable data management, analysis and visualization of semi-quantitative mass spectrometry based proteomics experiments. PIQMIe readily integrates peptide and (non-redundant) protein identifications and quantitations from multiple experiments with additional biological information on the protein entries, and makes the linked data available in the form of a light-weight relational database, which enables dedicated data analyses (e.g. in R) and user-driven queries. Using the web interface, users are presented with a concise summary of their proteomics experiments in numerical and graphical forms, as well as with a searchable protein grid and interactive visualization tools to aid in the rapid assessment of the experiments and in the identification of proteins of interest. The web server not only provides data access through a web interface but also supports programmatic access through RESTful web service. The web server is available at http://piqmie.semiqprot-emc.cloudlet.sara.nl or http://www.bioinformatics.nl/piqmie. This website is free and open to all users and there is no login requirement.

## INTRODUCTION

With recent technological advances in liquid chromatography–mass spectrometry (LC-MS) instrumentation, quantitation strategies and computational methods for MS data analysis, it has become possible to identify (to infer) and quantify thousands of proteins in a single shotgun proteomics experiment (1,2,3). Semi-quantitative MS-based proteomics relies on label-free approaches or on metabolic/chemical labeling of proteins—whereby at least one of the samples is enriched in stable heavy isotopes. In particular, the stable isotope labeling with amino acids in cell culture (SILAC) is a widely-used technique to interrogate the complex and dynamic nature of proteomes (4). In a typical SILAC proteome experiment, tens of thousands of peptides and thousands of (non-redundant) proteins are reliably identified and quantified from raw MS data, e.g. using the popular MaxQuant software (5) integrated with the Andromeda search engine (6). 

Further analyses of processed MS data, namely of those on peptide and protein (group) identifications and quantitations, are often facilitated by stand-alone, platform-specific spreadsheet tools including Microsoft Excel or dedicated Perseus software (http://www.perseus-framework.org). Although these tools are useful, they are not suitable for data management and integration, nor are scalable with increasing amounts of input data as compared to a database management or an information retrieval system (7). Common tasks such as summarizing or filtering peptide and protein lists for known contaminants and decoys (i.e. false positives inferred from the database search) involve manual steps that tend to be cumbersome and error-prone, and as such, impede accurate analysis and interpretation of results (8). Moreover, searching a long spreadsheet or large text file is computationally inefficient without a supporting index (sequential search). Complex queries that require joint data from separate peptide and protein (group) lists are not possible because the spreadsheet tools were not designed to model the relationships between different entities such as peptides, proteins and groups—as typically found in shotgun proteomics experiments (9). Although production-grade relational database management systems (RDBMS) such as the open-source MySQL, PostgreSQL or the commercial Oracle database enable efficient data management through the use of the structured query language (SQL), these require expertise to install and to configure a database server.

Several centralized repositories for MS-based proteomics have been developed in the past years, e.g. the Global Proteome Machine Database (GPMDB) (10), PeptideAtlas (11) and PRIDE (12), with the primary goal of providing a collection of peptide and/or protein identifications from multiple experiments. To our knowledge, MaxQB (13), ProteinCenter (Thermo Scientific) and QARIP (14) are the only available data management and/or web-based analysis platforms which can handle high-resolution semi-quantitative (SILAC) MS data processed by the MaxQuant software. However, MaxQB and ProteinCenter are closed-source solutions that cannot be freely used, deployed or modified by other proteomics laboratories, and QARIP is a web tool specifically developed for the analysis of regulated intramembrane proteolysis. The above issues motivated us to seek a light-weight, cross-platform and open-source solution that equips a proteomics researcher with a dedicated tool for data management, integration and analysis of peptide and protein lists obtained from the MaxQuant software.

We developed a descriptive web server, the Proteomics Identifications and Quantitations Data Management and Integration Service (PIQMIe) that aids in reliable management, analysis and visualization of semi-quantitative MS-based proteomics experiments. Importantly, PIQMIe does not aim at providing users with a complete proteomics workflow nor with a centralized proteomics repository but rather it focuses on the integration of peptide and (non-redundant) protein identifications and quantitations, as obtained from semi-quantitative MS data processed by the MaxQuant software, with additional biological information on the proteins from the UniProtKB database (15). Moreover, PIQMIe makes the results of the experiments more accessible through the web in the form of a light-weight and cross-platform SQLite database for user-driven queries and dedicated (off-line) statistical analyses on the locally stored database(s), e.g. using the R programming language (16). Furthermore, users are presented with a concise summary of their proteomics experiments (including replicates) in numerical and graphical forms. In addition, they are provided with a searchable protein grid and interactive visualization tools to facilitate rapid assessment of the experiments and identification of proteins of interest for the follow-up targeted assays. Finally, the web server not only provides data access through a web interface but also supports programmatic access through the RESTful JSON-based web service (17).

## MATERIALS AND METHODS

### Proteomics workflow

**Figure 1.**
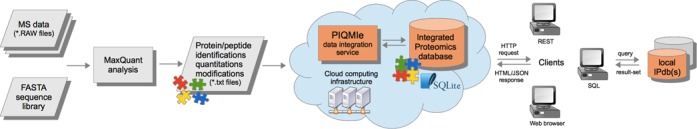
Computational proteomics workflow including the PIQMIe service. Before using the service, the semi-quantitative MS data are analyzed by the MaxQuant software. The resulting files, i.e. the peptide (‘evidence.txt’) and protein lists (‘proteinGroups.txt’) are uploaded together with the used FASTA sequence library to the server through the submission web page. PIQMIe then populates an SQLite database called the Integrated Proteomics database (IPdb), and makes the linked data accessible through (i) a local SQL interface, (ii) remote RESTful web service or (iii) a web browser.

The principle of using the PIQMIe service in a semi-quantitative proteomics workflow is illustrated in Figure 1. PIQMIe takes the results of the MS data processed by MaxQuant and makes them available on the web, as well as enables data access through different clients. In particular, the use of an SQL interface enables efficient retrieval of the data stored in the (local) SQLite database for dedicated data analyses using libraries implemented in domain-specific or general-purpose programming languages (e.g. DanteR (18) or Python Data Analysis Library, http://pandas.pydata.org).

During data submission, a user must provide a short description of the data set and select the MaxQuant result files including the FASTA sequence library of interest through the web form. PIQMIe then performs a preliminary client-side verification of the input files before the files are uploaded to the virtual server. Upon successful verification, the status of the submitted job is indicated with a progress bar and accompanied messages. The data processing might be unwillingly interrupted by an error raised at the server side, e.g. due to the use of a file with an unsupported or incorrect format. Once the processing of the job is successfully completed, the user receives a link with a unique job ID that enables private access to the result pages including the database file. The users’ data will be kept confidential and deleted from the web server after one week from the uploading date.

### Web server implementation and deployment

The PIQMIe server was developed using free and open-source components, in particular the CherryPy Python web application framework (version 3.2.2) bundled with the WSGI thread-pooled web server. The web server dynamically serves HTML5 web pages and data files in the JavaScript Object Notation (JSON) data-interchange format (text) and SQLite database file format (binary). Several scripts were written in Perl to parse the MaxQuant result files, i.e. the peptide list (‘vidence.txt’) and protein list (‘proteinGroups.txt’) for qualitative and quantitative data, as well as to extract additional biological information from the UniProtKB database, such as the primary sequences, species names, function annotations, protein evidence and gene symbols contained in a FASTA protein sequence library. Once the user's files are uploaded to the server and parsed, the resulting data files are imported into database tables populated *a priori* in SQLite (version 3.7.9), which is a self-contained, server-less and zero-configuration SQL database engine. Specifically, the database schema consists of several ‘core’ tables wherein peptide and protein (group) identifications and quantitations from multiple experiments (e.g. based on duplex or triplex SILAC) are readily stored and integrated with additional biological information on the protein entries including post-translational modifications. In addition, the schema includes several pre-defined queries on the tables (views) to ease, e.g. the filtering of contaminants and decoys, the preparation of peptide- and protein-level summaries or input data for the front-end visualization.

In order to provide a web application that scales dynamically with increasing user demands, we deployed the PIQMIe server, as a virtual machine (VM) installed with the Ubuntu Linux 12.04 LTS (64-bit) operating system, on the HPC Cloud computing infrastructure (using a customized version of the OpenNebula platform) operated by the Dutch national HPC and e-Science support center (SURFsara). In principle, the VM including the PIQMIe installation could be deployed on other academic or commercial clouds of type Infrastructure-as-a-Service such as the Amazon Elastic Compute Cloud (EC2), e.g. using the ‘cloud bursting’ feature of the infrastructure currently in use.

### Web interface implementation and layout

PIQMIe is equipped with a user-friendly graphical user interface that supports the most common web browsers such as Firefox, Google Chrome, Internet Explorer, Safari and Opera. For the front-end development, we used freely available components: the Bootstrap collection of HTML- and CSS-based design templates (version 3.0.0) and JavaScript libraries, such as jQuery (1.11.0), jqGrid (4.6), D3.js (3.3.6) and Google's JSAPI, for document manipulation and data-driven visualization of bar charts, peptide coverage map and 2D scatterplot. Users are provided with the option to save their dynamically generated graphics in vector and bitmap formats (i.e. in SVG, PDF and PNG).

The web site comprises the data submission or home page and the results page, each with different sections (tabs) shown in the top navigation bar. For example, the ‘Help’ tab links to additional documentation about the PIQMIe service and the ‘Sample Data’ tab links to the description of the proteomics study used as a test case, including the input/output files. The results page is organized into (i) the ‘Download’ section including a download link to the user's SQLite database, (ii) the summaries on ‘Peptides’, ‘Proteins’, ‘Protein Groups’ and ‘Regulated Proteins’ and (iii) interactive tools such as the ‘Search Grid’ and 2D ‘Scatterplot’ for user-driven queries and interactive data visualization.

### Sample data set

The practical use of the PIQMIe service is exemplified using a recently published SILAC-based proteomics study on bone formation and mineralization (19). The raw MS data were made available by the authors and re-analyzed using a newer version of the MaxQuant/Andromeda software (version 1.3.0.5) with the same parameter settings, except the use of a more recent human FASTA sequence library from the UniProtKB (release 2013_11) instead of the discontinued IPI database (20). In this study, the responses to activin A, a transforming growth factor-β superfamily member, on human mesenchymal stem cells (hMSC) derived osteoblast differentiation and mineralization were investigated using semi-quantitative MS-based proteomics with duplex SILAC metabolic labeling. Specifically, it involved a reciprocal labeling strategy in which both the activin A treated and control samples were cultured on light and heavy isotope-enriched culture media to obtain more reliable quantitative data compared to a single-experiment approach. The analysis focused on the protein composition and changes in the extracellular compartments, namely the extracellular matrix (ECM) and matrix vesicles (MVs) (Supplementary Table S1).

### Use case: comparative semi-quantitative proteomics study of human mesenchymal stem cells

#### Summarizing and reporting experiments

**Figure 2.**
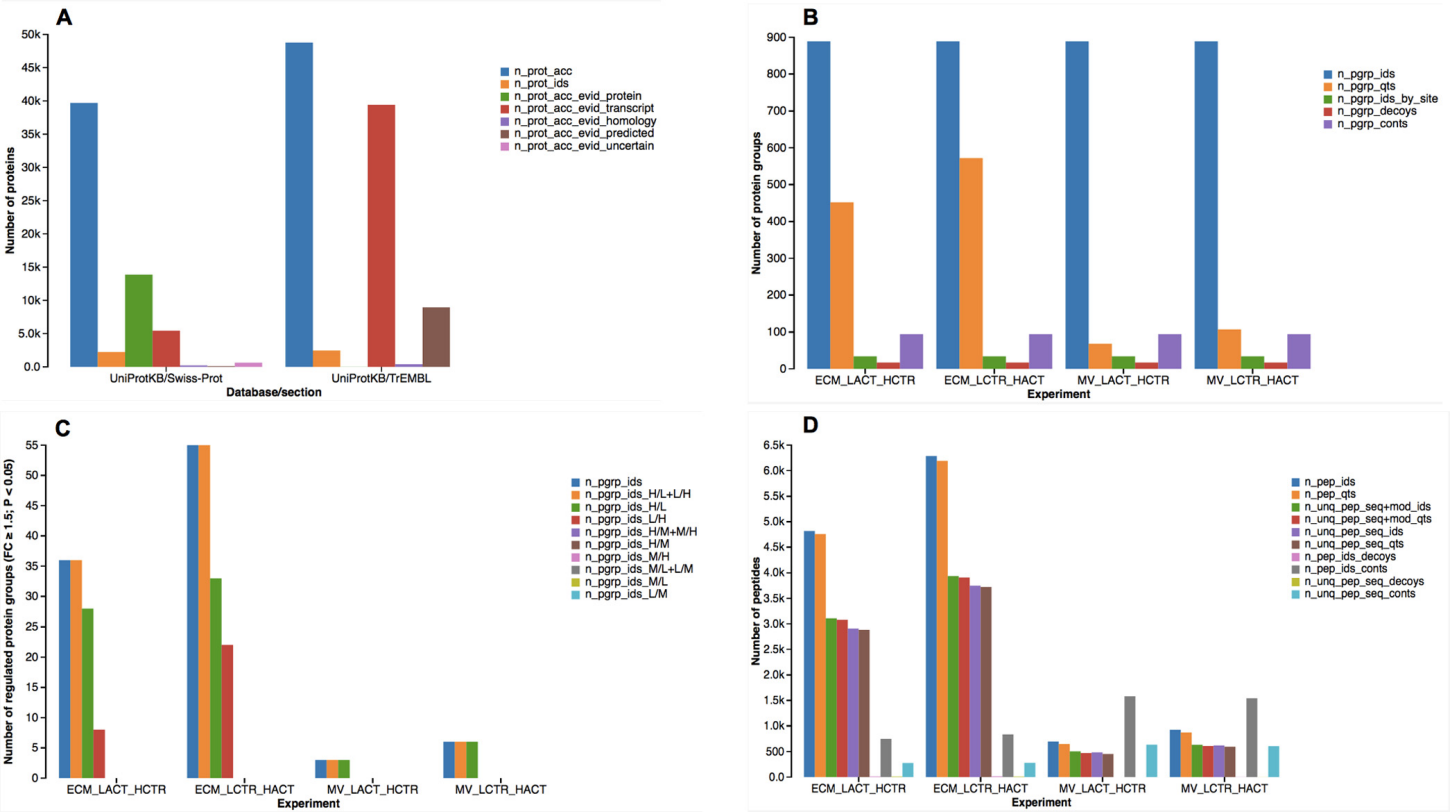
Overall summary of the SILAC ECM and MV experiments at the peptide and protein levels using bar charts: (**A**) database-dependent protein identifications; (**B**) non-redundant protein (groups) identifications and quantitations; (**C**) potentially regulated non-redundant proteins (FC ≥ 1.5; P value < 0.05); (D) peptide identifications and quantitations. Full description of the abbreviations used in the bar charts: *n_prot_acc*, number of protein accessions including isoforms in the source database (or FASTA sequence library); *n_prot_ids*, number of MS-based protein identifications including splice isoforms, filtered for decoys and contaminants; *n_prot_acc_evid_protein*, number of protein accessions with protein-level evidence; *n_prot_acc_evid_transcript*, number of accessions with transcript-level evidence; *n_prot_acc_evid_homology*, number of accessions with homology-based evidence; *n_prot_acc_evid_predicted*, number of accessions predicted *in silico*; *n_prot_acc_evid_uncertain*, number of accessions with uncertain evidence; *n_pgrp_ids*, number of non-redundant protein identifications, filtered for decoys and contaminants; *n_pgrp_qts*, number of non-redundant protein quantitations; *n_pgrp_ids_by_site*, number of non-redudant proteins identified by modification site; *n_pgrp_decoys*, number of non-redundant proteins detected as decoys (false positives); *n_pgrp_conts*, number of non-redundant proteins detected as contaminants; *n_pgrp_ids*, union of differentially regulated proteins identified in all conditions, filtered for decoys and contaminants; *n_pgrp_ids_H/L+L/H*, number of up- AND down-regulated proteins identified in both conditions H/L and L/H; *n_pgrp_ids_H/L*, number of up- OR down-regulated proteins identified in the H/L condition; *n_pgrp_ids_L/H*, number of up- OR down-regulated proteins identified in the L/H condition; *n_pep_ids*, number of redundant peptide identifications, filtered for decoys and contaminants; *n_pep_qts*, number of redundant peptide quantitations; *n_unq_pep_seq+mod_ids*, number of non-redundant peptide identifications unique by sequence and modifications; *n_unq_pep_seq+mod_qts*, number of non-redundant peptide quantitations unique by sequence and modifications; *n_unq_pep_seq_ids*, number of non-redundant peptide identifications unique by sequence; *n_unq_pep_seq_qts*, number of non-redundant peptide quantitations unique by sequence; *n_pep_ids_decoys*, number of redundant peptides detected as decoys (false positives); *n_pep_ids_conts*, number of redundant peptides detected as contaminants; *n_unq_pep_seq_decoys*, number of non-redundant peptide decoys unique by sequence; *n_unq_pep_seq_conts*, number of non-redundant peptide contaminants unique by sequence. For the exact values shown in the bar charts, refer to the tabulated data in the Supplementary Tables S2–S5.

The PIQMIe service processed the sample data in about a minute upon data upload and presented a concise summary of the SILAC experiments in numerical and graphical forms (Figure 2 and Supplementary Tables S2–S7). The pooled analysis of the ECM and MV proteomes resulted in the identification (inference) of 4693 proteins from the UniProtKB database, of which about half belong to the high-quality, manually curated UniProtKB/Swiss-Prot section (2238 entries). As this proteomics study is targeted rather than unbiased, the total number of proteins identified from the MS data is relatively low (about 5%) given the known human proteome, i.e. all human proteins verified experimentally and predicted *in silico* according to the UniProtKB database (88 473 proteins including splice isoforms, release 2013_11) (Supplementary Table S2). The identified proteins were clustered into 889 (non-redundant) protein groups, excluding those detected as contaminants and decoys (111 in total), by the MaxQuant software. More than half of the groups were associated with SILAC ratios (Heavy/Light) based on at least two peptide quantitation events in the ECM replicates, i.e. 572 groups (64%) versus 452 (51%). In terms of protein quantitations, the MV proteome was more limited than that of the ECM because fewer peptides were identified (up to 8-fold) from the low abundant MVs, i.e. 107 and 68 protein groups quantitated in the forward and reverse SILAC MV experiment, respectively. However, the total number of (unique) peptide contaminants was greater in the MV than in ECM experiments (Supplementary Tables S3 and S4). Proteins regulated by activin A signaling were identified per SILAC experiment by applying two types of cutoffs: (i) on the normalized protein ratios as fold-change (FC ≥ 1.5) and (ii) on the intensity-based significance B (P value < 0.05) corrected for multiple hypothesis testing with the Benjamini and Hochberg method (5). In total, 55/6 and 36/3 protein groups were found regulated in the forward and reverse SILAC ECM/MV experiment, respectively (Supplementary Table S5).

#### Interactive data visualization and query tools

**Figure 3.**
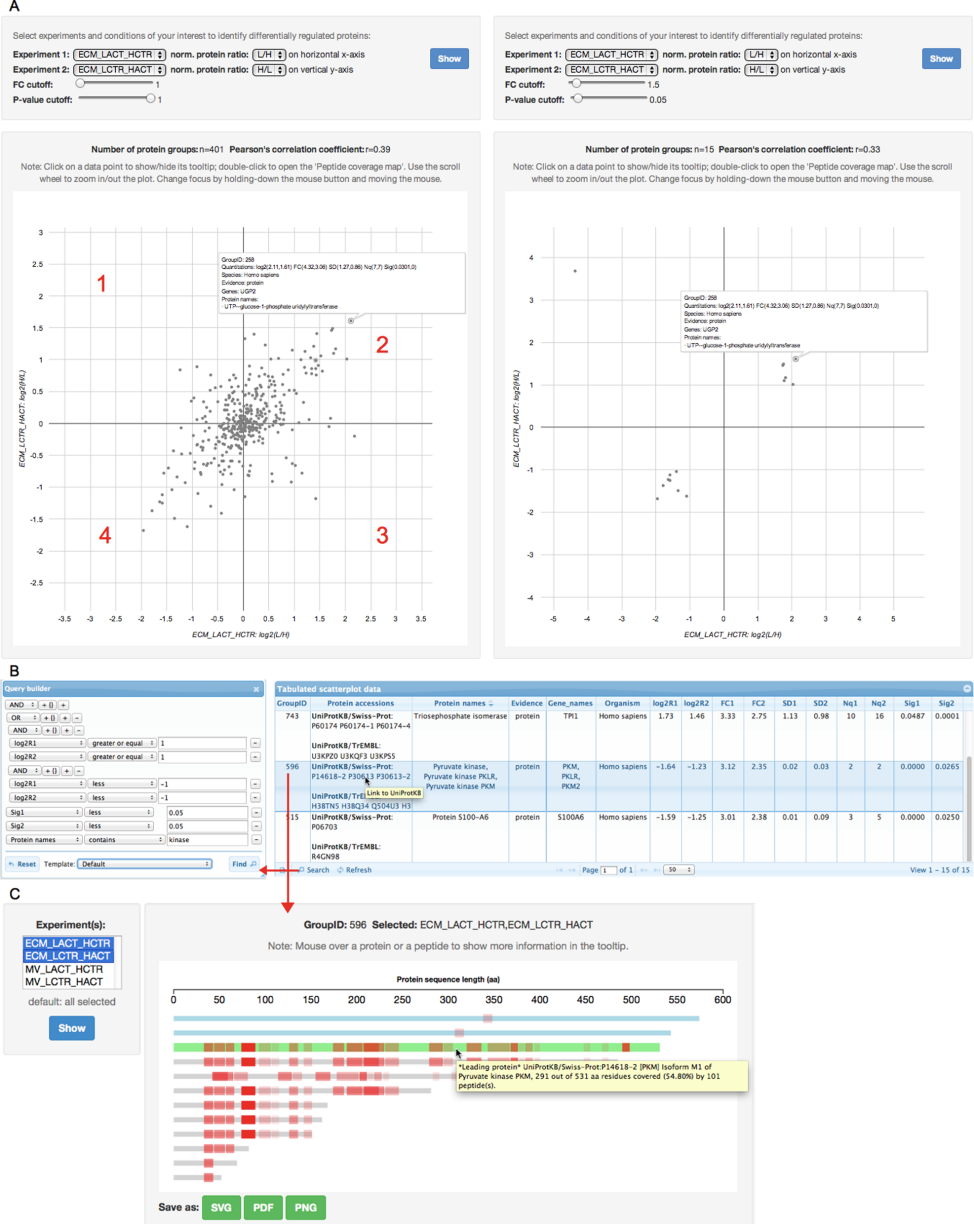
Interactive visualization and query tools available through the PIQMIe web interface. (**A**) 2D scatterplots of protein quantitations from reciprocal SILAC ECM experiments before (left figure) and after (right figure) the use of fold-change and intensity-based significance B cutoffs (FC ≥ 1.5; P value < 0.05). The plots are divided into four quadrants: the 1st and 3rd quadrants contain proteins that are inconsistently up- or down-regulated in the reciprocal experiments (false positives) whereas the 2nd and 4th quadrants contain proteins that are consistently up- and down-regulated by activin A signaling, respectively. For example, the UGP2 is consistently up-regulated in both ECM experiments. Moreover, the scatterplots are accompanied by the Pearson's correlation coefficient (*r*) computed for a pair of (reciprocal) SILAC experiments to aid in assessing the reproducibility of the replicate experiments. (**B**) Searchable protein grid with a query builder enables filtering of tabulated data by applying Boolean and/or relational operators on one or more columns of the grid. An example query is shown to select proteins with consistent (normalized) SILAC ratios from the set of potentially regulated proteins (FC ≥ 2; P value < 0.05) annotated as ‘kinase’. (**C**) Peptide coverage map shows the location and distribution of identified peptides (in red) within their parent proteins of a group. In the group (ID: 596), three protein entries belong to the manually curated UniProtKB/Swiss-Prot (in blue and green) including the ‘leading’ or best-scoring protein (accession: P14618-2, in green), as identified by the MaxQuant/Andromeda search, while the remaining nine proteins belong to the automatically annotated (unreviewed) UniProtKB/TrEMBL section (in gray). In addition, users can choose which experiments to view in the map. Note: All protein groups, accessions and peptides reported in the web interface are provided with hyperlinks to the appropriate site.

The PIQMIe web interface provides tools for searching and visualizing the results obtained from one or more proteomics experiments (or replicates). Specifically, one can use the searchable protein grid and the interactive 2D scatterplot with cut-off sliders, e.g. to detect proteins which are consistently up- or down-regulated in reciprocal SILAC experiments. (Figure 3A and B). Using this approach on the sample data set, we obtained a smaller set of regulated protein groups (FC ≥ 1.5; P value < 0.05), with 14 found in the ECM but none in the MV, compared to the single-experiment approach described above. We repeated this procedure by applying a less stringent filter on this data set, i.e. without the P value threshold as used in the original publication, which yielded 50 and 7 regulated protein groups in the ECM and the MV experiments, respectively (Supplementary Tables S6 and S7). Among the most regulated proteins in the ECM were those associated with glucose metabolism such as the UTP-glucose-1-phosphate uridylyltransferase (UGP2) and phosphoglucomutase-1 (PGM1). In the MV experiments, we found proteins involved in calcium flux in the MVs, of which annexin A4 was among the most down-regulated proteins by activin A signaling.

In shotgun proteomics experiments, it is common to obtain inferred protein groups rather than unambiguously identified proteins (singletons) because the peptide-centric approach results in one or more matching proteins (including their splice isoforms or paralogs) per peptide. Therefore, a representative or leading protein of each group is commonly selected as the best-scoring one with the maximum number of peptides identified but all other potential protein identifications are also kept. Depending on the FASTA sequence library against which the MS data were searched, the choice of the leading protein is crucial. For example, if the library originates from a database that contains annotations of distinct quality (e.g. the UniProtKB/Swiss-Prot *versus* UniProtKB/TrEMBL), reporting a poorly annotated protein (mainly from UniProtKB/TrEMBL) might complicate the interpretation of results. In the searchable protein grid, each protein group is reported with the highest protein evidence and a non-redundant set of function annotations (excluding duplicate or uninformative annotations such as ‘uncharacterized protein’ and ‘unknown protein’) based on all protein accessions rather than a single representative in that group, so that the most relevant information is presented to the users. In addition, the protein grid links each group with an integrated map that enables interactive visualization of peptides including their matching parent proteins as identified in the experiment(s) (Figure 3C).

## CONCLUSIONS AND PERSPECTIVES

In this article we presented PIQMIe, a cloud-based web application for reliable and scalable data management, analysis and visualization of semi-quantitative MS data processed by the popular MaxQuant software. PIQMIe provides users with a reporting tool that aids in the assessment of semi-quantitative shotgun proteomics experiments based on data properties summarized in numerical and graphical forms. We adapted a novel approach in managing and sharing the processed MS data using a light-weight RDBMS that does not require installation and configuration of a database server. In particular, PIQMIe transforms the user's input files into a single cross-platform database file contained with integrated peptide- and protein-level qualitative and quantitative data, and makes the resulting database available on the web for user-driven queries. This approach enables more efficient data access (using SQL) and analyses compared to the use of a plain text file or a spreadsheet. The web server also supports programmatic access through RESTful web service. Future work includes the implementation of proteomics data standards developed by the Proteomics Standards Initiative, in particular the mzIdentML and mzQuantML formats (21).

## SUPPLEMENTARY DATA

Supplementary Data are available at NAR Online.

Supplementary Data
