# Supplementary material for: PIQMIe: a web server for semi-quantitative proteomics data management and analysis
Source: Nucleic Acids Res. 2014 May 26;42(Web Server issue):W100–6. doi: 10.1093/nar/gku478 (PMC4086067; doi:10.1093/nar/gku478)
Supplement: Supplementary Data [file supp_42_W1_W100__index.html]

Supplementary Data 

# PIQMIe: a web server for semi-quantitative proteomics data management and analysis

## Supplementary Data

**Files in this Data Supplement:**

- Supplemental Tables
