# Supplementary material for: PIQMIe: a web server for semi-quantitative proteomics data management and analysis
Source: Nucleic Acids Res. 2014 May 26;42(Web Server issue):W100–6. doi: 10.1093/nar/gku478 (PMC4086067; doi:10.1093/nar/gku478)
Supplement: Supplementary Data [file supp_gku478_nar-00666-web-b-2014-File005.doc]

**Table S1.** Two-states (duplex) SILAC-based proteomics experiments on the extracellular matrix (ECM) and matrix vesicles (MVs).

| **Experiment name** | **'Light' sample**  (^12^C_6_,^14^N_2_-Lys and ^12^C_6_, ^14^N_4_-Arg) | **'Heavy' sample**  (^13^C_6_-Lys and ^13^C_6_, ^15^N_4_-Arg) |
| --- | --- | --- |
| ECM_LACT_HCTR | activin A | control |
| ECM_LCTR_LACT | control | activin A |
| MV_LACT_HCTR | activin A | control |
| MV_LCTR_HACT | control | activin A |

**Table S2.** Summary of database-dependent protein identifications. *db* – source database/section; *n_prot_acc* – number of protein accessions including isoforms in the source database (or FASTA sequence library); *n_prot_ids* – number of MS-based protein identifications including splice isoforms, filtered for decoys and contaminants; *n_prot_acc_evid_protein* – number of protein accessions with protein-level evidence; *n_prot_acc_evid_transcript* – number of protein accessions with transcript-level evidence; *n_prot_acc_evid_homology* – number of protein accessions with homology-based evidence; *n_prot_acc_evid_predicted* – number of proteins predicted *in silico*; *n_prot_acc_evid_uncertain* – number of protein accessions whose evidence is uncertain.

| db | n_prot_acc | n_prot_ids | n_prot_acc_evid_protein | n_prot_acc_evid_transcript | n_prot_acc_evid_homology | n_prot_acc_evid_predicted | n_prot_acc_evid_uncertain |
| --- | --- | --- | --- | --- | --- | --- | --- |
| UniProtKB/Swiss-Prot | 39697 | 2238 | 13870 | 5438 | 215 | 113 | 636 |
| UniProtKB/TrEMBL | 48776 | 2457 | 18 | 39412 | 398 | 8948 | 0 |

**Table S3.** Summary of non-redundant protein (group) identifications and quantitations. *exp_name* – experiment name; *n_pgrp_ids* – number of non-redundant protein identifications, filtered for decoys and contaminants; *n_pgrp_qts* – number of non-redundant protein quantitations; *n_pgrp_ids_by_site* – number of non-redudant proteins identified by modification site; *n_pgrp_decoys* – number of non-redundant proteins detected as decoys (false positives); *n_pgrp_conts* – number of non-redundant proteins detected as contaminants.

| exp_name | n_pgrp_ids | n_pgrp_qts | n_pgrp_ids_by_site | n_pgrp_decoys | n_pgrp_conts |
| --- | --- | --- | --- | --- | --- |
| ECM_LACT_HCTR | 889 | 452 | 34 | 17 | 94 |
| ECM_LCTR_HACT | 889 | 572 | 34 | 17 | 94 |
| MV_LACT_HCTR | 889 | 68 | 34 | 17 | 94 |
| MV_LCTR_HACT | 889 | 107 | 34 | 17 | 94 |

**Table S4.** Summary of peptide identifications and quantitations. *n_pep_ids* – number of redundant peptide identifications, filtered for decoys and contaminants; *n_pep_qts* – number of redundant peptide quantitations; *n_unq_pep_seq+mod_ids* – number of non-redundant peptide identifications unique by sequence and modifications; *n_unq_pep_seq+mod_qts* – number of non-redundant peptide quantitations unique by sequence and modifications; *n_unq_pep_seq_ids* - number of non-redundant peptide identifications unique by sequence; *n_unq_pep_seq_qts* - number of non-redundant peptide quantitations unique by sequence; *n_pep_ids_decoys* - number of redundant peptides detected as decoys (false positives); *n_pep_ids_conts* - number of redundant peptides detected as contaminants; *n_unq_pep_seq_decoys* - number of non-redundant peptide decoys unique by sequence; *n_unq_pep_seq_conts* - number of non-redundant peptide contaminants unique by sequence.

| exp_name | n_pep_ids | n_pep_qts | n_unq_pep_seq+mod_ids | n_unq_pep_seq+mod_qts | n_unq_pep_seq_ids | n_unq_pep_seq_qts | n_pep_ids_decoys | n_pep_ids_conts | n_unq_pep_seq_decoys | n_unq_pep_seq_conts |
| --- | --- | --- | --- | --- | --- | --- | --- | --- | --- | --- |
| ECM_LACT_HCTR | 4818 | 4759 | 3107 | 3079 | 2907 | 2883 | 12 | 748 | 11 | 276 |
| ECM_LCTR_HACT | 6286 | 6191 | 3936 | 3907 | 3748 | 3723 | 15 | 836 | 11 | 279 |
| MV_LACT_HCTR | 696 | 647 | 503 | 470 | 484 | 452 | 0 | 1581 | 0 | 634 |
| MV_LCTR_HACT | 926 | 873 | 632 | 608 | 618 | 594 | 3 | 1544 | 2 | 604 |

**Table S5.** Summary of potentially regulated non-redundant proteins per SILAC experiment. *n_pgrp_ids –* union of differentially regulated proteins identified in all conditions, filtered for decoys and contaminants; *n_pgrp_ids_H/L+L/H –* number of up- AND down-regulated proteins identified in both conditions H/L and L/H; *n_pgrp_ids_H/L –* number of up- OR down-regulated proteins identified in the H/L condition; *n_pgrp_ids_L/H –* number of up- OR down-regulated proteins identified in the L/H condition. Note: We show the values in *n_pgrp_ids* and *n_pgrp_ids_H/L+L/H* despite being always equal for duplex SILAC experiments but this is not necessarily the case for triplex SILAC experiments.

| exp_name | n_pgrp_ids | n_pgrp_ids_H/L+L/H | n_pgrp_ids_H/L | n_pgrp_ids_L/H |
| --- | --- | --- | --- | --- |
| ECM_LACT_HCTR | 36 | 36 | 28 | 8 |
| ECM_LCTR_HACT | 55 | 55 | 33 | 22 |
| MV_LACT_HCTR | 3 | 3 | 3 | 0 |
| MV_LCTR_HACT | 6 | 6 | 6 | 0 |

**Table S6.** List of proteins (groups) regulated by activin A signaling in the MVs. Numbers *1* and *2* in the column names refer to the *experiment 1* (MV_LACT_HCTR) and e*xperiment 2* (MV_LCTR_HACT), respectively; l*og2R1* – log2 of the normalized SILAC protein ratio 1 (L/H); *log2R2* – log2 of the normalized SILAC protein ratio 2 (H/L); *FC1* – fold-change of the protein ratio 1; *FC2* – fold-change of the protein ratio 2; *SD1* – standard deviation of the mean protein ratio 1; *SD2* – standard deviation of the mean protein ratio 2; *Nq1* – number of peptide quantitations used to estimate protein ratio 1; *Nq2* – number of peptide quantitations used to estimate protein ratio 2; *Sig1* – significance B of the protein ratio 1 (P-value); *Sig2* – significance B of the protein ratio 2 (P-value). Note: This list corresponds to the tabulated data shown below the interactive scatterplot and filtered for false positives (i.e. proteins with inconsistent reciprocal SILAC ratios) using FC ≥ 1.5 but no P-value cutoff.

| **GroupID** | **Protein accessions** | **Protein names** | **Evidence** | **Genes** | **Organism** | **log2R1** | **log2R2** | **FC1** | **FC2** | **SD1** | **SD2** | **Nq1** | **Nq2** | **Sig1** | **Sig2** |
| --- | --- | --- | --- | --- | --- | --- | --- | --- | --- | --- | --- | --- | --- | --- | --- |
| ≥ 1.5-fold DOWN-regulated by activin A | | | | | | | | | | | | | | | |
| [276](http://piqmie.semiqprot-emc.cloudlet.sara.nl/pepmap/a000000000000000000000000000000000000001?grp_id=276) | UniProtKB/Swiss-Prot: [P04216](http://www.uniprot.org/uniprot/P04216)  UniProtKB/TrEMBL: [E9PNQ8](http://www.uniprot.org/uniprot/E9PNQ8) [E9PIM6](http://www.uniprot.org/uniprot/E9PIM6) [J3QRJ3](http://www.uniprot.org/uniprot/J3QRJ3) | Thy-1 membrane glycoprotein | protein | THY1 | Homo sapiens | -1.13 | -0.99 | 2.19 | 1.98 | 0.12 | 0.29 | 3 | 4 | 0.7696 | 0.4619 |
| [490](http://piqmie.semiqprot-emc.cloudlet.sara.nl/pepmap/a000000000000000000000000000000000000001?grp_id=490) | UniProtKB/Swiss-Prot: [P02790](http://www.uniprot.org/uniprot/P02790) [Q5T013](http://www.uniprot.org/uniprot/Q5T013) [Q5T013-3](http://www.uniprot.org/uniprot/Q5T013-3) [Q5T013-4](http://www.uniprot.org/uniprot/Q5T013-4) [Q5T013-2](http://www.uniprot.org/uniprot/Q5T013-2)  UniProtKB/TrEMBL: [E7EWH8](http://www.uniprot.org/uniprot/E7EWH8) [J9JIE9](http://www.uniprot.org/uniprot/J9JIE9) [F6UJY9](http://www.uniprot.org/uniprot/F6UJY9) [H0YB70](http://www.uniprot.org/uniprot/H0YB70) [H0YB18](http://www.uniprot.org/uniprot/H0YB18) | Hemopexin, Putative hydroxypyruvate isomerase, Hydroxypyruvate isomerase | protein | HPX, HYI | Homo sapiens | -2.72 | -3.59 | 6.57 | 12.07 | 2.77 | 2.30 | 4 | 10 | 0.1286 | 0.2021 |
| [506](http://piqmie.semiqprot-emc.cloudlet.sara.nl/pepmap/a000000000000000000000000000000000000001?grp_id=506) | UniProtKB/Swiss-Prot: [P05106](http://www.uniprot.org/uniprot/P05106) [P05106-3](http://www.uniprot.org/uniprot/P05106-3) [P05106-2](http://www.uniprot.org/uniprot/P05106-2)  UniProtKB/TrEMBL: [H3BM21](http://www.uniprot.org/uniprot/H3BM21) [B4DTY9](http://www.uniprot.org/uniprot/B4DTY9) [I3L4X8](http://www.uniprot.org/uniprot/I3L4X8) | Integrin beta-3, Integrin beta | protein | ITGB3 | Homo sapiens | -1.16 | -2.74 | 2.23 | 6.66 | 1.88 | 1.91 | 5 | 4 | 0.7603 | 0.2343 |
| [553](http://piqmie.semiqprot-emc.cloudlet.sara.nl/pepmap/a000000000000000000000000000000000000001?grp_id=553) | UniProtKB/Swiss-Prot: [P09525](http://www.uniprot.org/uniprot/P09525)  UniProtKB/TrEMBL: [Q6P452](http://www.uniprot.org/uniprot/Q6P452) [B4DDF9](http://www.uniprot.org/uniprot/B4DDF9) | Annexin A4, Annexin | protein | ANXA4 | Homo sapiens | -2.09 | -3.87 | 4.27 | 14.60 | 0.36 | 1.96 | 2 | 10 | 0.3815 | 0.1957 |
| [566](http://piqmie.semiqprot-emc.cloudlet.sara.nl/pepmap/a000000000000000000000000000000000000001?grp_id=566) | UniProtKB/Swiss-Prot: [P10915](http://www.uniprot.org/uniprot/P10915)  UniProtKB/TrEMBL: [D6RBS](http://www.uniprot.org/uniprot/D6RBS1) [D6RG04](http://www.uniprot.org/uniprot/D6RG04) [D6RAK7](http://www.uniprot.org/uniprot/D6RAK7) [D6RBX9](http://www.uniprot.org/uniprot/D6RBX9) [D6RC59](http://www.uniprot.org/uniprot/D6RC59) | Hyaluronan and proteoglycan link protein 1 | transcript | HAPLN1 | Homo sapiens | -0.70 | -0.93 | 1.63 | 1.90 | 0.11 | 0.08 | 7 | 2 | 0.8916 | 0.4787 |
| ≥ 1.5-fold UP-regulated by activin A | | | | | | | | | | | | | | | |
| [635](http://piqmie.semiqprot-emc.cloudlet.sara.nl/pepmap/a000000000000000000000000000000000000001?grp_id=635) | UniProtKB/Swiss-Prot: [P23528](http://www.uniprot.org/uniprot/P23528)  UniProtKB/TrEMBL: [E9PK25](http://www.uniprot.org/uniprot/E9PK25) [E9PP50](http://www.uniprot.org/uniprot/E9PP50) [G3V1A4](http://www.uniprot.org/uniprot/G3V1A4) [E9PQB7](http://www.uniprot.org/uniprot/E9PQB7) [E9PS23](http://www.uniprot.org/uniprot/E9PS23) [E9PLJ3](http://www.uniprot.org/uniprot/E9PLJ3) | Cofilin-1, Cofilin 1 (Non-muscle) | protein | CFL1 | Homo sapiens | 0.92 | 1.33 | 1.89 | 2.52 | 0.74 | 0.19 | 3 | 4 | 0.4357 | 0.6567 |
| [530](http://piqmie.semiqprot-emc.cloudlet.sara.nl/pepmap/a000000000000000000000000000000000000001?grp_id=530) | UniProtKB/Swiss-Prot: [P07737](http://www.uniprot.org/uniprot/P07737)  UniProtKB/TrEMBL: [K7EJ44](http://www.uniprot.org/uniprot/K7EJ44) [I3L3D5](http://www.uniprot.org/uniprot/I3L3D5) | Profilin-1, Profilin 1 | protein | PFN1 | Homo sapiens | 1.61 | 1.02 | 3.05 | 2.02 | 1.66 | 0.29 | 2 | 2 | 0.2997 | 0.7707 |

**Table S7.** Top 10 list of proteins (groups) regulated by activin A signaling in the ECM. Numbers *1* and *2* in the column names refer to the *experiment 1* (ECM_LACT_HCTR) and e*xperiment 2* (ECM_LCTR_HACT), respectively; *log2R1* – log2 of the normalized SILAC protein ratio 1 (L/H); *log2R2* – log2 of the normalized SILAC protein ratio 2 (H/L); *FC1* – fold-change of the protein ratio 1; *FC2* – fold-change of the protein ratio 2; *SD1* – standard deviation of the mean protein ratio 1; *SD2* – standard deviation of the mean protein ratio 2; *Nq1* – number of peptide quantitations used to estimate protein ratio 1; *Nq2* – number of peptide quantitations used to estimate protein ratio 2; *Sig1* – significance B of the protein ratio 1 (P-value); *Sig2* – significance B of the protein ratio 2 (P-value). Note: This list is a subset of the tabulated data shown below the interactive scatterplot and filtered for false positives (i.e. protein groups with inconsistent reciprocal SILAC ratios) using FC ≥ 1.5 but no P-value cutoff. The table entries are sorted by the *Log2R1* column.

| **GroupID** | **Protein accessions** | **Protein names** | **Evidence** | **Genes** | **Organism** | **log2R1** | **log2R2** | **FC1** | **FC2** | **SD1** | **SD2** | **Nq1** | **Nq2** | **Sig1** | **Sig2** |
| --- | --- | --- | --- | --- | --- | --- | --- | --- | --- | --- | --- | --- | --- | --- | --- |
| ≥ 1.5-fold DOWN-regulated by activin A | | | | | | | | | | | | | | | |
| [486](http://piqmie.semiqprot-emc.cloudlet.sara.nl/pepmap/a000000000000000000000000000000000000001?grp_id=486) | UniProtKB/Swiss-Prot: [P02545](http://www.uniprot.org/uniprot/P02545) [P02545-2](http://www.uniprot.org/uniprot/P02545-2) [P02545-3](http://www.uniprot.org/uniprot/P02545-3) [P02545-4](http://www.uniprot.org/uniprot/P02545-4)  UniProtKB/TrEMBL: [Q6UYC3](http://www.uniprot.org/uniprot/Q6UYC3) [D6RAQ3](http://www.uniprot.org/uniprot/D6RAQ3) [Q5TCI8](http://www.uniprot.org/uniprot/Q5TCI8) [H0YAB0](http://www.uniprot.org/uniprot/H0YAB0) | Prelamin-A/C | protein | LMNA | Homo sapiens | -1.96 | -1.68 | 3.90 | 3.21 | 0.24 | 1.18 | 7 | 10 | 0.0000 | 0.0077 |
| [594](http://piqmie.semiqprot-emc.cloudlet.sara.nl/pepmap/a000000000000000000000000000000000000001?grp_id=594) | UniProtKB/Swiss-Prot: [P14174](http://www.uniprot.org/uniprot/P14174) | Macrophage migration inhibitory factor | protein | MIF | Homo sapiens | -1.79 | -1.37 | 3.47 | 2.59 | 0.07 | 0.04 | 4 | 3 | 0.0000 | 0.0176 |
| [596](http://piqmie.semiqprot-emc.cloudlet.sara.nl/pepmap/a000000000000000000000000000000000000001?grp_id=596) | UniProtKB/Swiss-Prot: [P14618-2](http://www.uniprot.org/uniprot/P14618-2) [P30613](http://www.uniprot.org/uniprot/P30613) [P30613-2](http://www.uniprot.org/uniprot/P30613-2)  UniProtKB/TrEMBL: [H3BTN5](http://www.uniprot.org/uniprot/H3BTN5) [H3BQ34](http://www.uniprot.org/uniprot/H3BQ34) [Q504U3](http://www.uniprot.org/uniprot/Q504U3) [H3BTJ2](http://www.uniprot.org/uniprot/H3BTJ2) [H3BUW1](http://www.uniprot.org/uniprot/H3BUW1) [H3BT25](http://www.uniprot.org/uniprot/H3BT25) [H3BU13](http://www.uniprot.org/uniprot/H3BU13) [H3BQZ3](http://www.uniprot.org/uniprot/H3BQZ3) [H3BN34](http://www.uniprot.org/uniprot/H3BN34) | Pyruvate kinase PKM, Pyruvate kinase PKLR, Pyruvate kinase | protein | PKM, PKLR, PKM2 | Homo sapiens | -1.64 | -1.23 | 3.12 | 2.35 | 0.02 | 0.03 | 2 | 2 | 0.0000 | 0.0265 |
| [515](http://piqmie.semiqprot-emc.cloudlet.sara.nl/pepmap/a000000000000000000000000000000000000001?grp_id=515) | UniProtKB/Swiss-Prot: [P06703](http://www.uniprot.org/uniprot/P06703)  UniProtKB/TrEMBL: [R4GN98](http://www.uniprot.org/uniprot/R4GN98) | Protein S100-A6 | protein | S100A6 | Homo sapiens | -1.59 | -1.25 | 3.01 | 2.38 | 0.01 | 0.09 | 3 | 5 | 0.0000 | 0.0250 |
| [831](http://piqmie.semiqprot-emc.cloudlet.sara.nl/pepmap/a000000000000000000000000000000000000001?grp_id=831) | UniProtKB/Swiss-Prot: [Q16555](http://www.uniprot.org/uniprot/Q16555) [Q16555-2](http://www.uniprot.org/uniprot/Q16555-2) [Q14194-2](http://www.uniprot.org/uniprot/Q14194-2) [Q14194](http://www.uniprot.org/uniprot/Q14194) [Q14195-2](http://www.uniprot.org/uniprot/Q14195-2) [Q14195](http://www.uniprot.org/uniprot/Q14195)  UniProtKB/TrEMBL: [E9PD68](http://www.uniprot.org/uniprot/E9PD68) [E5RFU4](http://www.uniprot.org/uniprot/E5RFU4) [H0YBT4](http://www.uniprot.org/uniprot/H0YBT4) [F5GWI3](http://www.uniprot.org/uniprot/F5GWI3) | Dihydropyrimidinase-related protein 2, Dihydropyrimidinase-related protein 1, Dihydropyrimidinase-related protein 3 | protein | DPYSL2, CRMP1, DPYSL3 | Homo sapiens | -1.59 | -1.12 | 3.00 | 2.17 | 1.25 | 0.97 | 6 | 8 | 0.0000 | 0.0374 |
| ≥ 1.5-fold UP-regulated by activin A | | | | | | | | | | | | | | | |
| [258](http://piqmie.semiqprot-emc.cloudlet.sara.nl/pepmap/a000000000000000000000000000000000000001?grp_id=258) | UniProtKB/Swiss-Prot: [Q16851](http://www.uniprot.org/uniprot/Q16851) [Q16851-2](http://www.uniprot.org/uniprot/Q16851-2)  UniProtKB/TrEMBL: [E7EUC7](http://www.uniprot.org/uniprot/E7EUC7) [C9JNZ1](http://www.uniprot.org/uniprot/C9JNZ1) [C9JQU9](http://www.uniprot.org/uniprot/C9JQU9) [C9JVG3](http://www.uniprot.org/uniprot/C9JVG3) [C9JWG0](http://www.uniprot.org/uniprot/C9JWG0) [C9JUW1](http://www.uniprot.org/uniprot/C9JUW1) [C9JTZ5](http://www.uniprot.org/uniprot/C9JTZ5) [F2Z3H1](http://www.uniprot.org/uniprot/F2Z3H1) | UTP--glucose-1-phosphate uridylyltransferase | protein | UGP2 | Homo sapiens | 2.11 | 1.61 | 4.32 | 3.06 | 1.27 | 0.86 | 7 | 7 | 0.0301 | 0.0000 |
| [683](http://piqmie.semiqprot-emc.cloudlet.sara.nl/pepmap/a000000000000000000000000000000000000001?grp_id=683) | UniProtKB/Swiss-Prot: [P36871](http://www.uniprot.org/uniprot/P36871) [P36871-2](http://www.uniprot.org/uniprot/P36871-2) [P36871-3](http://www.uniprot.org/uniprot/P36871-3) | Phosphoglucomutase-1 | protein | PGM1 | Homo sapiens | 2.03 | 1.01 | 4.09 | 2.02 | 1.58 | 1.14 | 3 | 12 | 0.0330 | 0.0252 |
| [964](http://piqmie.semiqprot-emc.cloudlet.sara.nl/pepmap/a000000000000000000000000000000000000001?grp_id=964) | UniProtKB/Swiss-Prot: [Q9Y281](http://www.uniprot.org/uniprot/Q9Y281) [Q9Y281-3](http://www.uniprot.org/uniprot/Q9Y281-3)  UniProtKB/TrEMBL: [F8WDN3](http://www.uniprot.org/uniprot/F8WDN3) | Cofilin-2 | protein | CFL2 | Homo sapiens | 1.81 | 1.17 | 3.51 | 2.26 | 0.19 | 0.22 | 3 | 2 | 0.0437 | 0.0058 |
| [128](http://piqmie.semiqprot-emc.cloudlet.sara.nl/pepmap/a000000000000000000000000000000000000001?grp_id=128) | UniProtKB/Swiss-Prot: [P58546](http://www.uniprot.org/uniprot/P58546)  UniProtKB/TrEMBL: [C9JL85](http://www.uniprot.org/uniprot/C9JL85) | Myotrophin | protein | MTPN | Homo sapiens | 1.77 | 1.10 | 3.41 | 2.14 | 0.39 | 0.16 | 3 | 3 | 0.0465 | 0.0121 |
| [671](http://piqmie.semiqprot-emc.cloudlet.sara.nl/pepmap/a000000000000000000000000000000000000001?grp_id=671) | UniProtKB/Swiss-Prot: [P31949](http://www.uniprot.org/uniprot/P31949) | Protein S100-A11 | protein | S100A11 | Homo sapiens | 1.75 | 1.49 | 3.37 | 2.81 | 0.03 | 1.27 | 2 | 4 | 0.0473 | 0.0001 |
